# Working life, health and well-being of parents: a joint effort to uncover hidden treasures in European birth cohorts

**DOI:** 10.5271/sjweh.3980

**Published:** 2021-09-30

**Authors:** Monica Ubalde-Lopez, Tina Garani-Papadatos, Ghislaine Scelo, Maribel Casas, Claudia Lissåker, Susan Peters, Ellen Aagaard Nohr, Maria Albin, Raquel Lucas, Kyriaki Papantoniou, Kinga Polańska, Cecilia H Ramlau-Hansen, Jelena Šarac, Jenny Selander, Helena Skröder, Elena Vasileiou, Manolis Kogevinas, Ute Bültmann, Ingrid Sivesind Mehlum, Milena Maule

**Affiliations:** ISGlobal, Barcelona, Spain; Pompeu Fabra University (UPF), Barcelona, Spain; Spanish Consortium for Research on Epidemiology and Public Health (CIBERESP), Madrid, Spain; Department of Public Health Policy, School of Public Health, University of West Attica, Athens, Greece; Cancer Epidemiology Unit, Department of Medical Sciences, University of Turin and CPO-Piemonte, Turin, Italy; Unit of Occupational Medicine, Institute of Environmental Medicine, Karolinska Institutet, Stockholm, Sweden; Institute for Risk Assessment Sciences, Utrecht University, Utrecht, The Netherlands; Research Unit for Gynaecology and Obstetrics, Department of Clinical Research, University of Southern Denmark, Odense, Denmark; EPIUnit (Epidemiology Research Unit), Instituto de Saúde Pública, Universidade do Porto, Porto, Portugal; Departamento de Ciências da Saúde Pública e Forenses e Educação Médica, Faculdade de Medicina, Universidade do Porto, Porto, Portugal; Department of Epidemiology, Center for Public Health, Medical University of Vienna, Vienna, Austria; Department of Environmental and Occupational Health Hazards, Nofer Institute of Occupational Medicine, Lodz, Poland; Department of Public Health, Research Unit for Epidemiology, Aarhus University, Aarhus, Denmark; Center for Applied Bioanthropology, Institute for Anthropological Research, Zagreb, Croatia; Laboratory of Hygiene and Environmental Protection, Medical School, Democritus University of Thrace, Alexandroupolis, Greece; Hospital del Mar Medical Research Institute (IMIM), Barcelona, Spain; Department of Health Sciences, Community and Occupational Medicine, University of Groningen, University Medical Center Groningen, Groningen, The Netherlands; Department of Occupational Medicine and Epidemiology, National Institute of Occupational Health (STAMI), Oslo, Norway; Department of Community Medicine and Global Health, Institute of Health and Society, Faculty of Medicine, University of Oslo, Oslo, Norway

**Keywords:** life-course, occupational epidemiology, pooled analysis

## Abstract

**Objective:**

Birth cohorts collect valuable and under-utilized information on employment and health of parents before and during pregnancy, at birth, and sometimes after birth. In this discussion paper, we examine how these data could be exploited to study the complex relationships and interactions between parenthood, work, and health among parents themselves.

**Methods:**

Using a web-based database of birth cohorts, we summarize information on maternal employment and health conditions and other potentially related variables in cohorts spread throughout Europe. This provided information on what data are available and could be used in future studies, and what was missing if specific questions are to be addressed, exploiting the opportunity to explore work–health associations across heterogenous geographical and social contexts.

**Results:**

We highlight the many potentialities provided by birth cohorts and identify gaps that need to be addressed to adopt a life-course approach and investigate topics specific to the peri-pregnancy period, such as psychosocial aspects. We address the technical difficulties implied by data harmonization and the ethical challenges related to the repurposing of data, and provide scientific, ecological and economic arguments in favor of improving the value of data already available as a result of a serious investment in human and material resources.

**Conclusions:**

There is a hidden treasure in birth cohorts that deserves to be brought out to study the relationships between employment and health among working parents in a time when the boundaries between work and life are being stretched more than ever before.

Work represents the primary means of obtaining economic resources essential for material needs and is also central to individual identity and social roles. There are many potential causal pathways between work, health, and well-being. Health affects work ability and work, in turn, can be beneficial for health and well-being but may also carry risks for mental and physical health. The nature, quantity and quality of work, and its social context should be considered in balancing beneficial and harmful effects (1, 2).

Under-employment, long-term unemployment, poor working conditions, and job insecurity negatively affect health, well-being and social cohesion. Younger people and women are a population group particularly vulnerable to adverse working conditions (3). Childbearing typically occurs during early adulthood, which is a period of general good health and well-being. However, pregnancy and first years of childcaring are physically and mentally demanding periods for young mothers and fathers, who often struggle to return to full-time work after parental leave with direct consequences such as delay in their career trajectories and sense of self-realization. National social security and welfare systems, as well as familial socioeconomic status, may modify the impact of employment and working conditions on the health and well-being of parents. Little research is available on the health consequences of having young children on families while participating in the workforce. Health conditions specific to this time period, such as sleep deprivation and stress, must be considered, as well as their potential long-term health consequences. To examine these type of questions, a large number of variables are needed, including work-related and family-level constructs and individual determinants of health, particularly lifestyle factors, such as alcohol and tobacco consumption, diet, and physical activity.

Many birth cohorts throughout Europe collect information on employment and health of parents (especially mothers) before and during pregnancy, at birth, and often at one or more follow-up examinations after delivery (www.birthcohorts.net). These records include a wealth of valuable data on working life and health of parents during these time windows, which are typically collected for measuring their potential effects on the health of the children (4–7) (figure 1a), or as confounders in the relation between a risk factor and the health of the children (8, 9) (figure 1b). However, with its inherent longitudinal dimension, the birth cohort setting has greater potential – including the investigation of the direct relationship between work and parents’ health (figure 1b) – to address questions related to the causal and intertwined relationships between work and health of parents also in relation to the children’s health and other exposures collected at different time points (figure 1c). The conceptual framework is exemplified in figure 1 using the causal diagrams notation, whereby arrows represent possible causal effects and assumptions are encoded by the direction and the absence of arrows (10).

**Figure 1:**
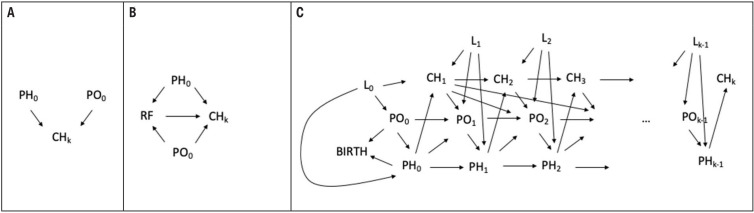
Conceptual framework for investigating causal relationships between work and health of parents also in relation to the children’s health. [PH_i_=parents’ health at time i (i=0: baseline, ie, during pregnancy or at birth); PO_i_=parents’ occupation at time i; RF=risk factor; CH_i_=child’s health at time i; L_i_=confounders at time i.] A. Occupation and health of parents at baseline (during pregnancy or at birth) as possible determinants of child’s health. B. Causal relationship between a risk factor and child’s health, controlling for occupation and health of parents at baseline. C. Possible more complex causal relationship between occupation and health of parents, and health of the child at different time points, including confounders.

In addition, birth cohorts represent a sizeable resource that would allow researchers to address specific domains difficult to assess in traditional occupational epidemiology studies, such as working life in relation to reproductive life and work-family conflicts.

In this discussion paper, we argue that parental work-related data collected in birth cohorts is a valuable but under-utilized resource that could be exploited more fruitfully in the collaboration between birth cohort research, occupational epidemiology and sociology. Existing birth cohort information as well the collection of new data on less studied aspects could then be used to their full potential to study the complex relationships and interactions between parenthood, work, and health in parents themselves.

For context, we first compare several indicators of welfare systems across countries in Europe. We then provide an overview of available data in existing European birth cohorts. Finally, we provide recommendations on how to overcome the methodological challenges that can arise when repurposing existing data usage.

## Working conditions: a cross-national comparison

Many factors can influence the health and well-being of working parents. Working conditions depend on a multitude of factors, often shaped by societal efforts in encouraging childbirth and participation in the workforce. Since World War II, a range of welfare systems have been developed in Europe to cover for childbearing and child care in an effort to promote both increasing birth rates and economic growth. Substantial differences exist between countries. We illustrate these differences through three examples: leave entitlements, childcare possibilities, and public spending towards family benefits.

*Leave entitlements*. In all European countries, working parents are entitled to a range of different leave types, the most common being maternity, paternity, and parental leaves (11). Maternity leave is intended to protect the health of the mother and new-born child in the period around childbirth. Paternity leave allows the father to spend time with the child and the mother in the period following childbirth. Parental leave is usually equally available to mothers and fathers and can take various shapes and follow different rules of transferability between parents and flexibility (eg, part-time work). It is a measure intended to provide both parents with the opportunity to spend time caring for a young child. Paid leave entitlements have increased in most European countries over the last decades and many countries have recently put leaves in place for fathers. Leave systems often show some level of flexibility, and international comparisons based on rigid criteria can be misleading. Nevertheless, clear between-country differences exist. For example, the length of postnatal leaves paid ≥75% of the reference income ranges from ≤14 weeks for the mother and ≤2 weeks for the father in several countries, such as France and Switzerland, to >50 weeks shareable between parents in Bulgaria and Sweden (11). Within countries, access and benefits provided vary depending on a broad number of factors, such as the number of siblings, employment in the public or private sector, minimum time employed prior to leave, the proportion of the reference salary returned (eg, one can sometimes chose to get a lower allowance but stay on leave a longer time).

**Figure 2:**
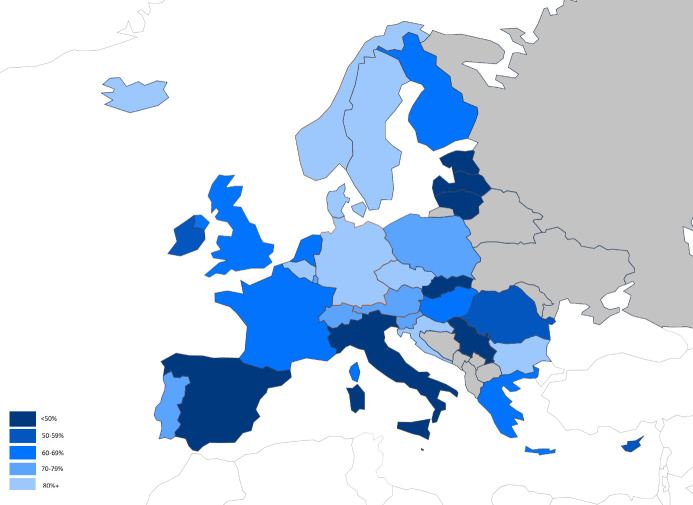
Proportion of households that are satisfied with the offer of formal childcare for their children aged 12 or below across 32 European countries (2016). Satisfaction is defined as not using more of the offer because they have no need of it, as opposed to, for example, because of financial reasons, lack of available places, not suitable opening hours, low quality of service, or inconvenient distance from household. Source: ref (5).

*Childcare possibilities*. Formal childcare is defined as care organized by a private or public structure (eg, centre-based day care, organized family day care, or qualified childminders organizations). Childcare is one of the 20 key principles of the European Pillar of Social Rights, and all European countries offer some type of formal childcare, although with considerable variations in availability and affordability. A survey conducted in 2016 by the European Commission concluded that 39% of children aged ≤12 years in the European Union (EU) receive formal childcare services, and 68% of the households are satisfied with the access to these services, whether they use them or not (12). The map depicted in figure 2 illustrates the heterogeneity in reported satisfaction with those services in the 27 EU countries, as well as the United Kingdom, Norway, Iceland, and Switzerland. The level of satisfaction ranged from <50–>80%. The reasons for not using more of the childcare services differed by country, the most cited being the lack of availability, the cost of the services, and distance from home. These difficulties may create work–family conflicts and are likely to have a large impact on the well-being of parents.

**Figure 3:**
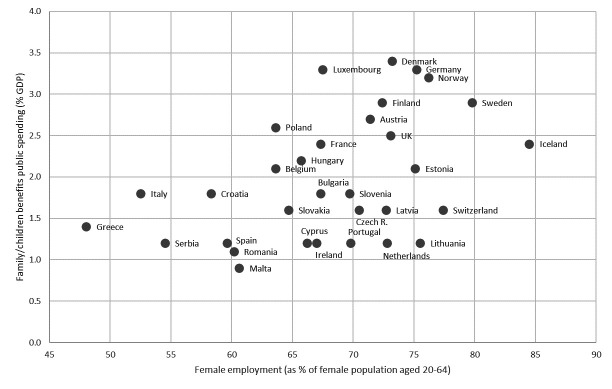
Female employment versus public spending on family/children benefits across 32 European countries in 2017. Family/children benefits spending refers to public spending and financial support targeting families and children. It includes cash transfers to families with children, public spending on services for families with children, and financial support for families provided through the tax system. The indicator is measured as a percentage of gross domestic product (GDP). [r=Pearson correlation coefficient weighted on the country population sizes]. Sources: Public spending on family benefits (2017): https://ec.europa.eu/eurostat/statistics-explained/index.php?title=Social_protection_statistics_-_family_and_children_benefits&oldid=483072. Employment statistics source (2017): https://ec.europa.eu/eurostat/statistics-explained/index.php?title=Employment_-_annual_statistics. Population sizes (2017): http://www.gapminder.org/.

*Women’s labor market participation and public spending towards family benefits*. Work participation among women started increasing at different time points in the mid-20^th^ century across Europe and proceeded at different rates in each country. Broad policy configurations that emerged after World War II might have contributed to these differences (13, 14). Figure 3 shows a plot of the proportion of women aged 20–64 years who were employed compared with the public spending on family/children benefits (as a percentage of country-specific gross domestic product) for 32 European countries in 2017. Again, we observe considerable variation in both indicators, with an apparent positive correlation. While the correlation (Pearson correlation coefficient weighted on the country population sizes: 0.62) may not imply any causal relationship, it does illustrate the heterogeneity that exists within Europe.

The differences observed in welfare systems across countries offer a unique opportunity to explore the association between work-related exposures and health of parents of young children across specific national contexts. However, such analyses can only be done within longitudinal studies that collect a broad range of individual-level variables, in addition to information on national welfare systems.

## Uniqueness and specificity of birth cohort data

Life-course studies with prospectively collected data constitute a valuable resource to investigate the role of work on health since they eliminate many biases related to retrospective or cross-sectional study designs and often provide information on numerous confounders at the individual level. Exposures (both occupational and environmental and related to lifestyle and health) experienced around the time of birth of a child could be overlooked by occupational epidemiology studies collecting the whole occupational and medical history of individuals and might be prone to recall biases if collected after years or even decades. The life-course theoretical model applied to epidemiology investigates the interactions between biological and social changes and their influence on health over time, accounting for the timing of multiple exposures and outcomes, and reflecting the impact that early exposure may have on later life. In occupational epidemiology, a life-course approach emphasizes how working life and the social context affect the relationships between work and health (figure 1c). This framework conceptualizes the changing nature of work as a life course experience in which the effect of working life transitions on future health, and conversely, the impact of health status on future working life, depend on place and time. Birth cohort studies represent a potentially important but yet underutilized resource to study the complex interplay between work, parenthood and parents’ health and well-being. To date, little is known about the interdependence of work and health among parents. To disentangle these complex relationships and understand their interdependence better, a life-course perspective to work and health within different labor markets and social security contexts is needed (15). Birth cohorts may be the right place to study, for example, transitions in and out of work (eg, maternity or paternity leave) and evaluate the impact of career interruption or the reduction of working hours on health.

During the last decades, many birth cohorts have been established in Europe, including both multi-­purpose ones and others specifically designed to investigate selected exposures or outcomes. In recent years, several EU-funded projects (ENRIECO – Environmental Health Risks in European Birth Cohorts; CHICOS – Developing a Child Cohort Research Strategy for Europe; EUCCONET – European Child Cohort Network; BRIDGE Health – Health Bridging Information and Data Generation for Evidence-based Health Policy and Research) have ben carried out to increase collaboration between cohorts and exploit their full potential, and others are currently underway [LifeCycle – Early Life Stressors and Lifecycle Health (16); I4C – The International Childhood Cancer Cohort Consortium (17)]. Recently, the EU Cost-Action OMEGA-NET developed a searchable web-based inventory of European cohorts with data on occupational exposures that partly includes birth cohorts (18, 19). Within the CHICOS project, a web-based database focused on birth cohorts (www.birthcohorts.net) originally established in 2005 within the European programme ChildrenGenoNetwork, was redesigned and updated. The database was developed as a tool to facilitate the exchange of knowledge and collaboration between cohorts and researchers. This website contains detailed information on social and environmental characteristics of children and their parents, parental and child health conditions, and biological samples collected at repeated time points throughout pregnancy and childhood, and is open for registration of cohorts worldwide (20). More recently, and under the umbrella of the LifeCycle project, some of these cohorts have undertaken a thoughtful harmonization of main variables to facilitate cross-cohort studies (16). Existing birth cohorts have mainly been used to study early-life determinants of child health and development, including maternal occupation [eg, refs (21, 22)]; however, they contain valuable information on employment and health of parents which has not been fully exploited.

To illustrate the data available in birth cohort studies, we have summarized information on maternal employment and health conditions and other potentially related variables in European cohorts with at least some occupational information (employment status or job title or occupational exposures) registered in www.birthcohorts.net (table 1 and supplementary table S1, www.sjweh.fi/article/3980). Although some of this information may not be up to date, the table provides an indication of what is available in these cohorts and can potentially be used in future studies. A total of 59 of the 103 European birth cohorts identified in www.birthcohorts.net contained some standard occupation-related information, such as employment status (N=30, eg, employed, unemployed, inactive, student), job title (N=23, usually classified according to the International Standard Classification of Occupations, 1988 version), and chemical occupational exposures (N=44, eg, pesticides, paints, radiation). Few cohorts have gathered information on heavy lifting (N=25) and working hours (N=11), and very few on work address (N=4), which can be linked with spatial data in a geographical information system (GIS) and provide information on the built and social environments. Using birth cohort data for investigating parental working life does come with drawbacks. Since, with some exceptions [eg, (23)], most cohorts were not set up to study parental working life, little has been collected on psychosocial factors and employment information has often only been collected at one time point, particularly during pregnancy but not afterwards. Consequently, there is poor information on parental occupational trajectories. Additionally, not many cohorts have data on family composition and family functioning, and adverse life events, such as deaths or job loss, which can have a huge impact on the parents’ and child’s health. Finally, the employment information has mostly been collected from the mother and, less frequently, from the father, opening potential problems such as a different validity of information about mothers and fathers or the legitimate use of data provided by third parties (when mothers have answered about father’s variables).

**Table 1:** Selected available information related to mothers’ work and socioeconomic status in 59 European birth cohorts (as reported in www.birthcohorts.net catalogue, accessed on 5 Feb 2021).

Cohort	General variables				Occupational exposures variables				
	
	Education	Income	Single parenthood	Employment status	Job title	Occupational exposures	Heavy lifts	Work hours	Work address
ABC	x	x				x			
ABCD	x	x	x	x	x	x	x	x	
ALSPAC	x					x	x		
BaBi	x	x	x	x	x	x	x		
BABIP	x	x	x	x	x				
Babycarecohort	x	x	x			x	x		
BAMSE	x			x	x				
BASIC	x	x	x	x					
BIB	x	x	x			x			
CELSPAC: TNG	x	x	x	x	x	x	x	x	x
CHOP	x		x	x	x			x	
COLLAGE	x	x				x			
Co.N.ER						x			
CRIBS	x	x	x	x	x	x	x		x
Czech Early Childhood Health						x			
DNBC	x					x	x		
ECLIPSES	x	x	x	x	x				
EDEN	x	x	x	x	x				
ELFE	x					x	x		
ELSPAC	x			x		x	x		
FCOU	x					x	x		
FLEHS 1 RefNb	x	x		x	x	x			
FLEHS 2 Ref Nb	x	x	x	x	x	x			
FLEHS III	x	x	x	x	x	x			
GASPII	x		x			x			
GECKO	x	x	x		x				
Generation R	x	x	x			x	x		
GISA	x	x		x	x	x	x		x
HbgBC	x		x	x					
HELMi	x		x	x					
HUMIS	x	x	x			x			
INMA	x			x		x	x		
INUENDO	x					x			
KANC	x		x			x			
KOALA	x					x			
Krakow	x		x			x			
KuBiCo	x		x	x	x				
Lifelines NEXT	x	x	x	x	x	x	x	x	
Lifeways	x	x	x				x		
LiNA	x	x	x	x				x	
LoewenKIDS	x	x	x	x	x				
LucKi	x			x	x	x		x	
Mamma & Bambino	x		x	x	x				
MoBa	x		x			x	x		
MUBICOS						x			
NEHO	x	x	x	x	x	x	x	x	x
NINFEA	x			x	x	x		x	
Odense	x		x			x			
PCB cohort	x		x			x			
PÉLAGIE	x	x	x			x	x		
Piccolipiù	x		x			x	x		
PLASTICITY			x			x	x		
Predict	x		x	x		x			
PRIDE	x	x	x	x	x	x	x	x	
REPRO_PL	x	x	x	x	x	x	x	x	
RHEA	x		x			x			
SWS	x		x	x	x		x	x	
Trieste						x			
WHISTLER	x	x	x			x	x		

Regarding health conditions and potential determinants (table 2), many cohorts have information on parental, mainly maternal, anthropometric measures (N=54), cardiovascular diseases (N=38), diabetes (N=46), psychological distress or mental health (n=48), respiratory health (N=26), and musculoskeletal diseases (N=27). Despite its relevance and appropriateness in the birth cohorts context, sleep disturbances were collected in a small number of cohorts (N=7). Regarding confounders and other health-related variables, all cohorts have collected information on active smoking (N=59), and many on alcohol consumption (N=55); fewer cohorts had collected data on substance use (N=33) and physical activity (N=42). The timing when these variables were collected varies by study and not all cohorts have repeatedly collected them over time. This is a limitation that will need to be overcome to perform large-scale longitudinal analysis of the effect of work on health. In several countries, however, and under some conditions (such as specific consent, provided by parents at the time of enrolment), cohort data may be linked with registries on education, income, employment, and social transfer payments, as well as registries on medication and health care utilization, including hospitalizations. This option may represent a solution to the lack of repeated collections of information currently limiting the use of birth cohorts that collected parental work and health-related variables at only one time point.

**Table 2:** Selected mothers’ health conditions and determinants collected in 59 European birth cohorts (as reported in www.birthcohorts.net catalogue, accessed on 5 Feb 2021).

Cohort	Anthro-pometry	Cardio-vascular diseases	Diabetes	Psycho-logical distress	Mental health	Respiratory health	Musculo-skeletal diseases	Sleep disturbance	Active smoking	Alcohol consumption	Substance use	Physical activity
ABC	x		x		x				x	x	x	
ABCD	x	x	x	x	x	x	x	x	x	x	x	x
ALSPAC	x	x	x		x		x		x	x	x	x
BaBi	x	x	x	x		x			x	x	x	x
BABIP	x	x	x	x	x	x		x	x	x	x	x
Babycarecohort	x	x	x	x	x		x		x	x	x	x
BAMSE		x	x			x			x			
BASIC	x	x	x	x	x		x		x	x	x	
BIB	x	x	x	x	x		x		x	x		x
CELSPAC	x			x		x	x		x	x	x	x
CHOP	x	x		x	x	x			x	x		x
COLLAGE	x	x	x	x			x		x	x	x	x
Co.N.ER	x	x	x						x	x		
CRIBS	x	x	x	x	x	x	x		x	x	x	x
Czech Early Childhood Health	x	x	x						x			
DNBC	x	x	x		x		x		x	x	x	x
ECLIPSES	x			x	x				x	x	x	x
EDEN	x	x	x	x	x				x	x	x	x
ELFE	x	x	x	x	x		x		x	x		x
ELSPAC	x	x	x	x		x	x		x	x	x	x
FCOU	x	x	x	x	x		x		x	x	x	x
FLEHS 1 RefNb	x				x	x			x	x		
FLEHS 2 Ref Nb	x			x		x			x	x	x	x
FLEHS III	x			x		x			x	x	x	x
GASPII		x	x	x	x				x	x		
GECKO	x		x		x	x			x	x	x	x
Generation R			x	x	x		x		x	x	x	x
GISA	x	x	x	x		x			x	x	x	x
HgbBC	x					x	x		x			
HELMi	x	x	x	x		x			x	x		x
HUMIS	x		x	x	x				x	x	x	x
INMA	x	x	x	x	x				x	x	x	x
INUENDO	x		x						x	x		
KANC	x	x	x	x					x	x		
KOALA	x	x	x	x	x		x		x	x		x
Krakow	x								x	x	x	
KuBiCo	x	x	x	x	x	x	x	x	x	x	x	x
Lifelines NEXT	x	x	x	x	x	x	x	x	x	x	x	x
Lifeways	x		x	x	x				x	x	x	x
LiNA						x	x		x	x		
LoewenKIDS						x	x		x	x		
LucKi	x	x	x			x	x		x	x		x
Mamma & Bambino	x								x	x		x
MoBa	x	x	x	x	x	x	x		x	x	x	x
MUBICOS	x	x	x		x		x		x		x	
NEHO	x	x		x	x	x		x	x	x	x	x
NINFEA	x	x	x		x	x	x	x	x	x	x	x
Odense	x		x	x	x				x	x	x	x
PCB cohort	x								x	x	
PÉLAGIE	x		x						x	x		x
Piccolipiù	x	x	x	x	x		x		x	x		x
PLASTICITY	x		x		x				x			
Predict	x	x	x		x		x		x	x	x	x
PRIDE	x	x	x	x	x	x	x	x	x	x	x	x
REPRO_PL	x	x	x	x		x			x	x	x	x
RHEA	x		x	x	x				x	x		x
SWS	x	x	x	x	x	x			x	x		x
Trieste	x	x	x		x		x		x	x		
WHISTLER	x	x	x	x	x		x		x	x	x	x

Despite current limitations, collaborative studies using harmonized data from different birth cohorts will move the field on employment and health of working parents forward, allowing the study of the interplay between work participation and the health of the children, mothers and, in many circumstances, fathers. The identification of neglected topics and underdeveloped areas of investigation would be one of the first outputs of a synergy between birth cohort research and occupational epidemiology. As an example, our survey on existing information showed that the study of the effects of sleep deprivation on health and work participation of parents of newborns would be difficult given the small number of birth cohorts that collected information on sleep problems. Social epidemiology applications would also be possible, such as examining the social mobility over time and determining its impact on health, and disentangling social causation and social selection processes.

## Technical challenges

General challenges in pooling cohort data, that are not specific to birth cohorts, may vary with each research question and include defining the target population, defining key covariates, and determining an analysis plan (24). Data harmonization is a crucial step before any pooled analyses can be conducted. Variables may have been recorded or measured differently, at various levels of detail, or may measure slightly different aspects of a certain exposure, outcome, as well as covariates (24). The workload of the harmonization step should not be underestimated (25) and a well-defined codebook is essential. The complexity of harmonization varies per variable but needs considerable decision-making steps. Harmonization of key variables may lead to loss of information when going to a less detailed level or to missing fields when choosing a more detailed level (24). The resulting loss of information may, however, be counterbalanced by a larger sample size.

By treating pooled data as if they came from one sample, significant heterogeneity across studies may lead to misleading summary effect estimates. On the other hand, the heterogeneity of study participants in a pooled analysis may result in a better representation of the key target populations than in single studies (24). Further, analyses of comparatively rare occupational exposures and outcomes in individual studies may be statistically underpowered, but power can be increased by pooling data from several birth cohorts.

Many birth cohorts will only have few or no individual level data on occupational exposures. However, when job titles are available or cohorts can be linked to census data with job histories, occupational exposures can be estimated with job-exposure matrices (JEM) (26). A JEM is an efficient method to assess systematically a wide range of exposures in large study populations. Particularly when pooling data, this allows for standardized exposure assessment within and between studies. A general limitation of JEM is the ignorance of heterogeneity within jobs, while exposure may vary between workers, as well as within workers over time. A specific challenge for assessing occupational exposures in relation to adverse birth effects is the timing of the exposure. JEM assign exposures to a certain job, the information of which is typically available by calendar year. Due to this rather crude assessment, typically no distinction in exposure levels relative to the conception, gestation period and birth can be made with a JEM. Birth cohorts could provide valuable complementing information with specific job descriptions and their changes during these relevant windows of exposure.

## Ethical challenges

In many (although not all) instances, at the moment of enrolment in a birth cohort, parents consent to provide data about themselves and their child with the specific aim of studying the child health outcomes. Whereas the technical challenges mentioned above affect to some extent all pooled analyses, this implies that using birth cohort data to study parents’ health involves the ethical, legal and social implications of repurposing (secondary use) of data, ie, their use beyond that intended at the time of collection, including direct information extraction and possibly linkage with other datasets such as health records.

The notion of ‘hidden treasures’ mentioned in the title reflects the increasing realization that data are a valuable resource with an extendable range of potential uses. Maximizing the use of existing data and enhancing their interoperability can benefit research, study participants and society as a whole, on the basis of scientific, economic, and ecological arguments. From a scientific point of view, our proposal would allow the investigation of risk factors and outcomes typically understudied in occupational epidemiology (such as work–life conflicts) adopting a life-course approach (15), as detailed above. As far as the economic impact of secondary use of data is concerned, economy of scale and saving of funds emerge as solid reasons for exhausting the value of existing data rather than requiring participants to provide new data or recruiting new participants. In the increasing discussions about the proper prioritization of the research agenda, a main recommendation posits that investment in additional research should always be preceded by systematic assessment of existing evidence (27). Thus, improving the value of data that are already available as a result of a serious investment in both human and material resources seems to be in line with a justified maximization of benefit and an incremental value of data. With regard to a disproportionate effort in recontacting cohort participants to request consent for further use of their data, cost is considered by the GDPR (Recital 26) as an objective factor in the process of “identification, taking into consideration the available technology at the time of the processing and technological developments”. Thus, an ecological argument towards the optimal use and statistical power of data that have already been collected could be evoked here: repurposing existing resources implies higher research efficiency, interoperability of data, and reduction of waste by circumventing the collection of new data (28). Capitalizing on and deriving insight from existing data, instead of pursuing additional collection of information, may constitute a long-term resource for occupational epidemiology that would enhance the potential benefit of core resources.

The main ethical challenges related to repurposing of data concern proper access to existing data, processing and sharing through special agreements and approvals from research ethics committees, if and when required. Sharing, in particular, is also related to perceptions about how different research cultures affect the use and interpretation of ambiguous terms such as privacy of individuals, families, or groups, a factor which has to be further explored (29, 30). For example, in relation to the privacy of oneself and one’s child, studies reveal that parents show greater reluctance towards sharing child identifiers compared to their own (19). Attitudes also differ with regard to the type of data at stake, ie, biological compared to non-biological data: there seems to be a more liberal approach towards sharing non-biological data. Moreover, people seem to be more willing to share their data with academia than with the private sector (31).

Consent remains a key notion for the collation and processing of personal identifiable data, however the GDPR adopts a balanced approach between protection of personal data and enhancement of a European research area in the public interest. Therefore, for the use of data that were collected from a previous research project, details regarding the initial data collection and the remits of the informed consent are key to evaluating whether a new consent is necessary or not for further use of data. However, even when consent cannot be used as a lawful basis for processing given the high threshold set by the GDPR, the Regulation adopts the concept of compatibility of purpose: according to the general principle of Article 5, “further processing for archiving purposes in the public interest, scientific or historical research purposes or statistical purposes shall, in accordance with Article 89 (1), not be considered to be incompatible with the initial purposes (‘purpose limitation’)”. This presumption of compatibility has to take into account a number of key factors and safeguards, including technical and organizational measures to ensure respect for the principle of data minimization and to protect the subjects’ fundamental rights (Article 6 (4), Recital 50 of the GDPR).

An important and favorable characteristic of birth cohorts is that much effort is spent to keep a continuous relationship and contact with the participants in subsequent follow-ups for many years. This may act as a positive factor towards obtaining the necessary consents and integrating existing information with specific questions towards this new area of research, nevertheless, possible methodological and legal ramifications may arise.

## Concluding remarks

Birth cohorts have largely contributed to the understanding of the determinants of children’s health, including the role of maternal and paternal occupational exposures. We propose to evaluate the relationship between parental peri-pregnancy/perinatal occupation-related conditions and their health around and after the birth of their children. We also recommend to explore the potential interplay between parental occupation, parental health and children’s health.

Several previous EU projects have invested enormous efforts and resources in standardizing and harmonizing data of a wide variety of cohorts, created a comprehensive birth cohort inventory and illustrated that (i) data can be shared, combined, pooled and compared; and (ii) different studies may be complementary.

In this discussion paper, we argue that (i) birth cohorts that have collected parental occupational data can and should use them for purposes beyond the study of children’s health; (ii) birth cohorts that did not collect parental occupational data could consider starting to collect them; (iii) both should look into the possibility of expanding the data collected to include emerging topics in occupational health, including those specific to families such as work–family balance, as well as more cross-cutting issues (eg, ageing workforce, work trajectories, work as part of the exposome).

Birth cohorts have much potential in studying the relationships between work and health of parents, including the possibility to use this information trans-generationally and investigate their joint effect on the health of children. To further exploit their longitudinal nature to its full extent and address new research and societal issues, further collections of information on work and health trajectories or linkages with existing registries could be envisaged, establishing new contacts with cohort participants and renewing and extending their initial expression of consent.

Future birth cohorts or collection of information in existing ones may also consider including structural information on social protection and career-building to study the potential effects of parenthood on work, health and well-being. For example, partial contributions towards retirement benefits, interrupted careers, lower job quality and fewer skill upgrades may imply health and social disadvantages and even poverty later in life. Special attention could be given to psychosocial work factors, such as work–family balance/interference, as boundaries of work have become weaker. Recent shifts towards parents’ smart/telework and children’s distance learning determined by the COVID-19 pandemic may have shaken such boundaries even more. Little is known about the unmeasured effects of taking work home, and it is likely that the outsized share of household and childcare responsibilities carried by mothers has escalated (32). With their open gaze on the household, birth cohorts could be the most suitable approach to explore these research questions.

To further utilize the great potential for collaborative analyses, adequate funding – eventually at the EU level – is required and should be applied to boost future research on the intersection of employment and health among working parents.

## Supplementary material

Supplementary material

## References

[1] Flint E, Bartley M, Shelton N, Sacker A (2013). Do labour market status transitions predict changes in psychological well-being?. J Epidemiol Community Health.

[2] Waddell G, Burton KA (2006). Is work good for your health and well-being?[Internet].

[3] (2013). European Foundation for the Improvement of Living and Working Conditions. Working conditions of young entrants to the labor market.

[4] Pape K, Liu X, Sejbæk CS, Andersson NW, Larsen AD, Bay H (2021). Maternal life and work stressors during pregnancy and asthma in offspring. Int J Epidemiol.

[5] Pape K, Svanes C, Sejbæk CS, Malinovschi A, Benediktsdottir B, Forsberg B (2021). Parental occupational exposure pre- and post-conception and development of asthma in offspring. Int J Epidemiol.

[6] Sejbaek CS, Niclasen J, Bonde JP, Kristensen P, Larsen AD, Schlünssen V (2020). Maternal exposure to psychosocial job strain during pregnancy and behavioral problems in the 11-year-old children: a Danish cohort study. Eur Child Adolesc Psychiatry.

[7] Larsen AD, Schlünssen V, Christensen BH, Bonde JP, Obel C, Thulstrup AM (2014). Exposure to psychosocial job strain during pregnancy and odds of asthma and atopic dermatitis among 7-year old children - a prospective cohort study. Scand J Work Environ Health.

[8] Morales E, Romieu I, Guerra S, Ballester F, Rebagliato M, Vioque J (2012). INMA Project. Maternal vitamin D status in pregnancy and risk of lower respiratory tract infections, wheezing, and asthma in offspring. Epidemiology.

[9] Mahedy L, Hammerton G, Teyhan A, Edwards AC, Kendler KS, Moore SC (2017). Parental alcohol use and risk of behavioral and emotional problems in offspring. PLoS One.

[10] Greenland S, Pearl J, Robins JM (1999). Causal diagrams for epidemiologic research. Epidemiology.

[11] Koslowski A, Blum S, Dobrotić I, Kaufman G, Moss P (2020). 16th International Review on Leave Policies and Related Research [Internet]. Hagen: FernUniversität in Hagen.

[12] (2018). Eurostat News Release 30/2018. Access to social services: Almost 4 in 10 children in the EU receive formal childcare services [Internet]. Eurostat Press Office.

[13] Pettit B, Hook J (2005). The structure of women's employment in comparative perspective. Soc Forces.

[14] Kenjoh E (2005). New Mothers'Employment and Public Policy in the UK, Germany, the Netherlands, Sweden, and Japan. Labour.

[15] Amick BC, McLeod CB, Bültmann U (2016). Labor markets and health: an integrated life course perspective. Scand J Work Environ Health.

[16] Jaddoe VW, Felix JF, Andersen AN, Charles MA, Chatzi L, Corpeleijn E, LifeCycle Project Group (2020). The LifeCycle Project-EU Child Cohort Network: a federated analysis infrastructure and harmonized data of more than 250,000 children and parents. Eur J Epidemiol.

[17] Tikellis G, Dwyer T, Paltiel O, Phillips GS, Lemeshow S, Golding J (2018). International Childhood Cancer Cohort Consortium. The International Childhood Cancer Cohort Consortium (I4C): A research platform of prospective cohorts for studying the aetiology of childhood cancers. Paediatr Perinat Epidemiol.

[18] Turner MC, Mehlum IS (2018). Greater coordination and harmonisation of European occupational cohorts is needed. Occup Environ Med.

[19] Kogevinas M, Schlünssen V, Mehlum IS, Turner MC (2020). The OMEGA-NET International Inventory of Occupational Cohorts. Ann Work Expo Health.

[20] Larsen PS, Kamper-Jørgensen M, Adamson A, Barros H, Bonde JP, Brescianini S (2013). Pregnancy and birth cohort resources in europe: a large opportunity for aetiological child health research. Paediatr Perinat Epidemiol.

[21] Casas M, Cordier S, Martínez D, Barros H, Bonde JP, Burdorf A (2015). Maternal occupation during pregnancy, birth weight, and length of gestation: combined analysis of 13 European birth cohorts. Scand J Work Environ Health.

[22] Birks L, Casas M, Garcia AM, Alexander J, Barros H, Bergström A (2016). Regina Gražulevičienė. Occupational Exposure to Endocrine-Disrupting Chemicals and Birth Weight and Length of Gestation: A European Meta-Analysis. Environ Health Perspect.

[23] Hauge LJ, Kornstad T, Nes RB, Kristensen P, Irgens LM, Eskedal LT (2013). The impact of a child's special health care needs on maternal work participation during early motherhood. Paediatr Perinat Epidemiol.

[24] Lesko CR, Jacobson LP, Althoff KN, Abraham AG, Gange SJ, Moore RD (2018). Collaborative, pooled and harmonized study designs for epidemiologic research: challenges and opportunities. Int J Epidemiol.

[25] Swerdlow AJ, Harvey CE, Milne RL, Pottinger CA, Vachon CM, Wilkens LR (2018). The National Cancer Institute Cohort Consortium. The National Cancer Institute Cohort Consortium: An International Pooling Collaboration of 58 Cohorts from 20 Countries. Cancer Epidemiol Biomarkers Prev.

[26] Peters S (2020). Although a valuable method in occupational epidemiology, job-exposure -matrices are no magic fix. Scand J Work Environ Health.

[27] Chalmers I, Bracken MB, Djulbegovic B, Garattini S, Grant J, Gülmezoglu AM (2014). How to increase value and reduce waste when research priorities are set. Lancet.

[28] Doubal FN, Ali M, Batty GD, Charidimou A, Eriksdotter M, Hofmann-Apitius M (2017). Big data and data repurposing - using existing data to answer new questions in vascular dementia research. BMC Neurol.

[29] Shen Y (2016). Research Data Sharing and Reuse Practices of Academic Faculty Researchers: A Study of the Virginia Tech Data Landscape. Int J Digit Curation.

[30] Whittlestone J, Nyrup R, Alexandrova A, Dihal K, Cave S (2019). Ethical and societal implications of algorithms, data, and artificial intelligence: a roadmap for research.

[31] Dodd SX, Manhas KP, Page S, Letourneau N, Cui X, Tough S (2017). Governance and Privacy in a Provincial Data Repository - A Cross-sectional Analysis of Longitudinal Birth Cohort Parent Participants'Perspectives on Sharing Adult Vs. Child Research Data: Proceedings of the 6th International Conference on Data Science, Technology and Applications [Internet]. Madrid: SCITEPRESS - Science and Technology Publications. http://www.scitepress.org/DigitalLibrary/Link.aspx?doi=10.5220/0006430802080215.

[32] Heggeness ML (2020). Estimating the immediate impact of the COVID-19 shock on parental attachment to the labor market and the double bind of mothers. Rev Econ Househ.

