# Supplementary material for: Working life, health and well-being of parents: a joint effort to uncover hidden treasures in European birth cohorts
Source: Scand J Work Environ Health. 2021 Sep 30;47(7):550–60. doi: 10.5271/sjweh.3980 (PMC8504161; doi:10.5271/sjweh.3980)
Supplement: Supplementary material [file SJWEH-47-550-S001.pdf]

Working life, health and well-being of parents: a joint effort to uncover hidden treasures in European birth cohorts[1]

by Monica Ubalde-Lopez, PhD, Tina Garani-Papadatos, PhD, Ghislaine Scelo PhD,[5] Maribel Casas, PhD, Claudia Lissåker, PhD, Susan Peters, PhD, Ellen Aagaard Nohr, PhD, Maria Albin, PhD, Raquel Lucas, PhD, Kyriaki Papantoniou, PhD, Kinga Polańska, PhD, Cecilia H Ramlau-Hansen, PhD, Jelena Šarac, PhD, Jenny Selander, PhD, Helena Skröder, PhD, Elena Vasileiou, MSc, Manolis Kogevinas, PhD, Ute Bültmann, PhD, Ingrid Sivesind Mehlum, PhD, Milena Maule, PhD [2]

1. *Supplementary table*
2. *Correspondence to: Prof. Milena Maule, Cancer Epidemiology Unit, University of Turin, via Santena 7, 10126 Torino, Italy. [E-mail: milena.maule@unito.it*

Table S1 European birth cohorts with information on maternal occupation (Source: www.birthcohorts.net, accessed on 5 Feb 2021)

Cohort	*Full name*	*Country*	*Enrolment period*	*Mothers recruited*
1. ABC	Aarhus Birth Cohort	Denmark	1990-	100000
2. ABCD	Amsterdam Born Children and their Development	Netherlands	2003-2004	8266
3. ALSPAC	Avon Longitudinal Study of Parents & Children/Children of the 90s	United Kingdom	1990-1992	14000
4. BaBi	BaBi study, Bielefeld Birth cohort study	Germany	2013-2017	970
5. BABIP	Bogazici Mother-Baby Relationship Project	Turkey	2018-	150
6. Babycarecohort	BabyCare	Germany	NA	26
7. BAMSE	Children (Barn), Allergy, Milieu, Stockholm, Epidemiological study	Sweden	1994-1996	4089
8. BASIC	Biology, Affect, Stress, Imaging and Cognition	Sweden	2009-2019	6387
9. BIB	Born in Bradford	United Kingdom	2007-2010	14000
10. CELSPAC: TNG	Central European Longitudinal Studies of Parents and Children: The Next Generation	Czech Republic	2015-	500
11. CHOP	Childhood Obesity - Early Programming by Infant Nutrition	Belgium, Germany, Italy, Poland, Spain	2002-2004	1678
12. COLLAGE	COLLAGE 2014 Siberia	Russia	2014-	2803
13. Co.N.ER	Bologna Birth Cohort	Italy	2004-2005	654
14. CRIBS	Croatian Islands' Birth Cohort Study	Croatia	2015-2018	500
15. Czech Early Childhood Health	Czech Early Childhood Health	Czech Republic	1994-1999	7522
16. DNBC	Danish National Birth Cohort	Denmark	1996-2002	100418
17. ECLIPSES	ECLIPSES	Spain	2013-2018	700

18.	EDEN-France	Study on the pre and early postnatal determinants of child health and development	France	2003-2005	2000
19.	ELFE	Etude Longitudinale Française depuis l'Enfance	France	2011	20000
20.	ELSPAC	European Longitudinal Study of Pregnancy and Childhood	Czech Republic	1991-1992	5151
21.	FCOU	Family and Children of Ukraine	Ukraine	1992-1996	4510
22.	FLEHS 1 RefNb	FLemish Environment and Health Study 1- Reference Newborns	Belgium	2002-2004	1196
23.	FLEHS 2 Ref Nb	FLemish Environment and Health Study 2 Reference Newborn	Belgium	2008-2009	255
24.	FLEHS III	FLemish Environment and Health Study 3 Reference Newborns	Belgium	2013-2014	281
25.	GASPII	Genetic and Environment: Prospective Study on Infancy in Italy	Italy	2003-2004	693
26.	GECKO	GECKO Drenthe cohort	Netherlands	2006-2007	2997
27.	Generation R	Generation R	Netherlands	2002-2006	9778
28.	GISA	Gestão Integrada Saúde e Ambiente	Portugal	2007-2010	1645
29.	HbgBC	Helsingborg Birth Cohort 1964-1967	Sweden	1964-1967	4982
30.	HELMi	Health and Early Microbiota	Finland	2016-2018	1063
31.	HUMIS	Norwegian Human Milk Study	Norway	2003-2009	2500
32.	INMA	Environment and Childhood Project	Spain	1997-2008	3944
33.	INUENDO	Human Fertility at Risk from Biopersistent Organochlorines in the Environments	Greenland, Sweden, Poland, Ukraine	2002-2004	2269
34.	KANC	Kaunas cohort	Lithuania	2007-2009	4329
35.	KOALA	KOALA Birth Cohort Study	Netherlands	2000-2002	2900
36.	Krakow	Krakow Cohort	Poland	2000-2003	528
37.	KuBiCo	Kuopio Birth Cohort	Finland	2012-	4700
38.	Lifelines NEXT	Lifelines NEXT	Netherlands	2016-	N/A
39.	Lifeways	Lifeways Cross-Generation Cohort Study	Ireland	2001-2003	1061
40.	LiNA	Lifestyle and environmental factors and their Influence on Newborns Allergy risk	Germany	2006-2008	622
41.	LoewenKIDS	LoewenKIDS	Germany	2015-2018	783
42.	LucKi	LucKi	Netherlands	2006-	5000
43.	Mamma & Bambino	Mamma & Bambino	Italy	2014-	411
44.	MoBa	Norwegian Mother and Child Cohort Study	Norway	1999-2008	108500
45.	MUBICOS	Multiple Births Cohort Study	Italy	2009-	1000
46.	NEHO	Neonatal Environment and Health Outcomes	Italy	2018-2020	860
47.	NINFEA	Birth and Infancy: Effects of the Environment	Italy	2005-2016	6832
48.	Odense	Odense Child Cohort	Denmark	2010-2012	2553
49.	PCB cohort	Slovak PCB study	Slovakia	2002-2004	1134

50.	PÉLAGIE	Endocrine disruptors: longitudinal study on pregnancy abnormalities, infertility, and childhood	France	2002-2005	4000
51.	Piccolipiù	Piccolipiù	Italy	2011-2015	3338
52.	PLASTICITY	Life long follow-up of cognitive ability after birth risks	Finland	1971-	22359
53.	Predict	Rotterdam Periconception Cohort	Netherlands	2010-	1500
54.	PRIDE	Pregnancy and Infant Development Study	Netherlands	2011-2019	3200
55.	REPRO_PL	Polish Mother and Child Cohort Study	Poland	2007-2011	1800
56.	RHEA	Mother Child Cohort in Crete	Greece	2007-2008	1590
57.	SWS	Southampton Women's Survey	United Kingdom	1998-2002	3158
58.	Trieste	Trieste child development cohort	Italy	2007-2009	900
59.	WHISTLER	Wheezing Illnesses Study in LEidsche Rijn	Netherlands	2003-2011	1000
